# Supplementary figures and images for: KitBase Expanded: An Integrated Genomic and Phenotypic Resource for 3,268 Fast-Neutron-Irradiated Rice Mutants
Source: Database (Oxford). 2026 Jun 12;2026:baag024. doi: 10.1093/database/baag024 (PMC13262976; doi:10.1093/database/baag024)

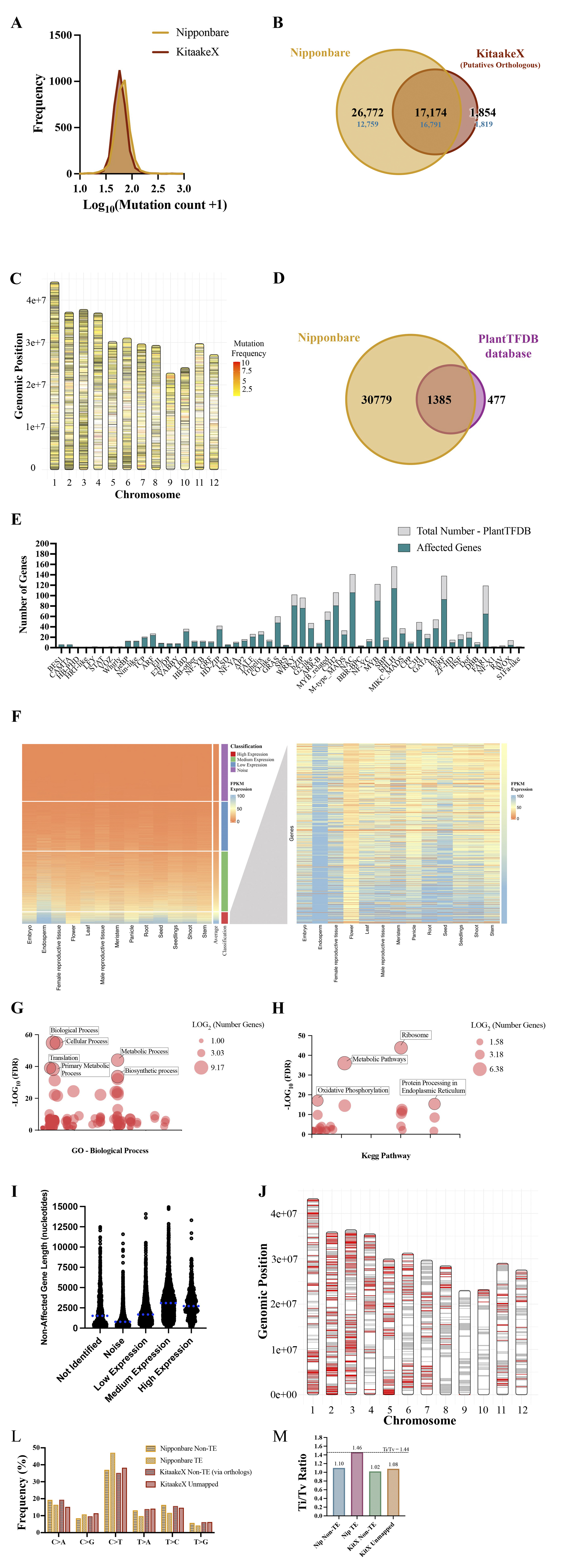

Supplement: baag024_Supplemental_Files [file baag024_supplemental_files.zip › Supplementary_Figure_1_New.jpg]

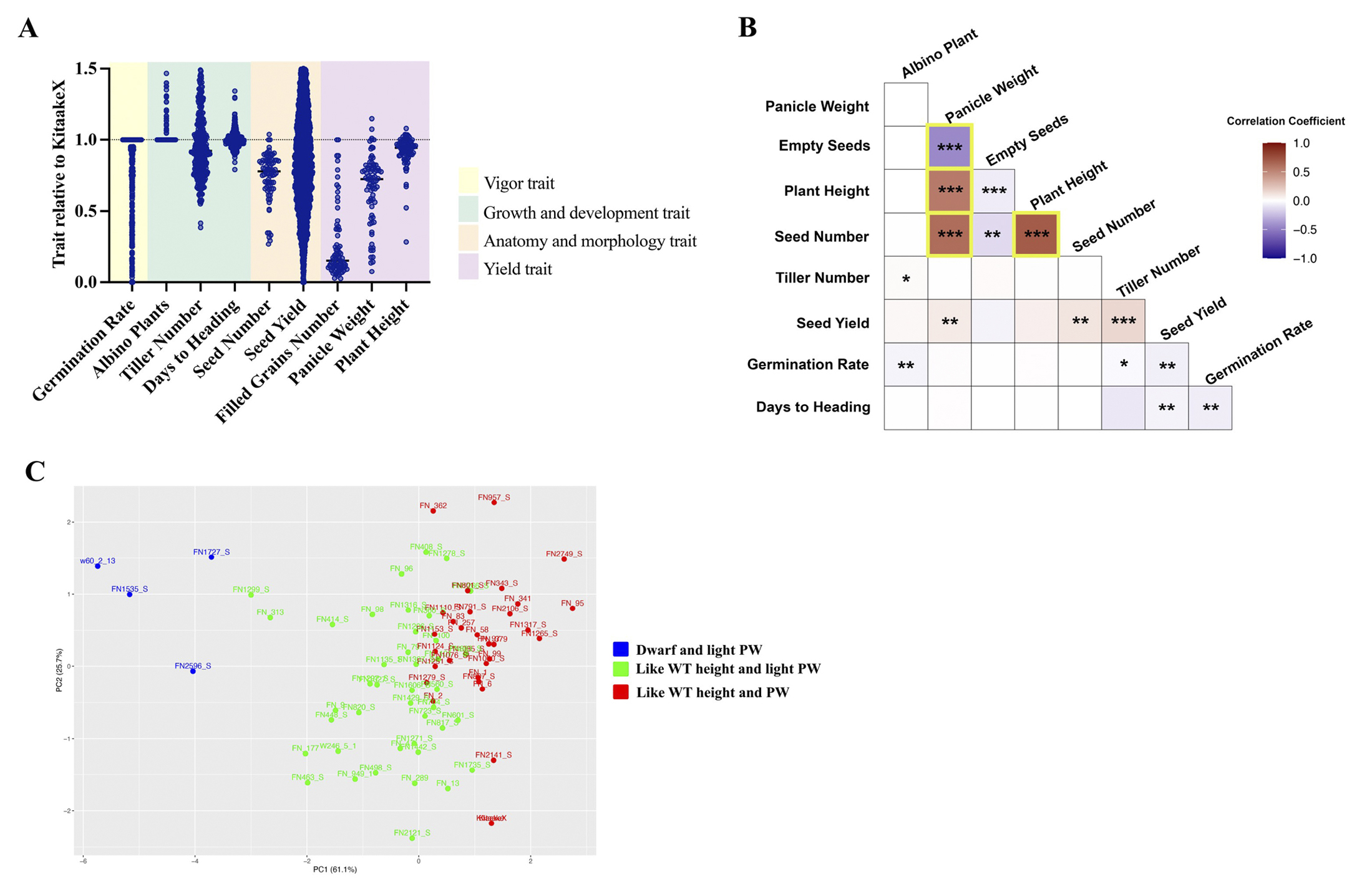

Supplement: baag024_Supplemental_Files [file baag024_supplemental_files.zip › Supplementary_Figure_2.jpg]

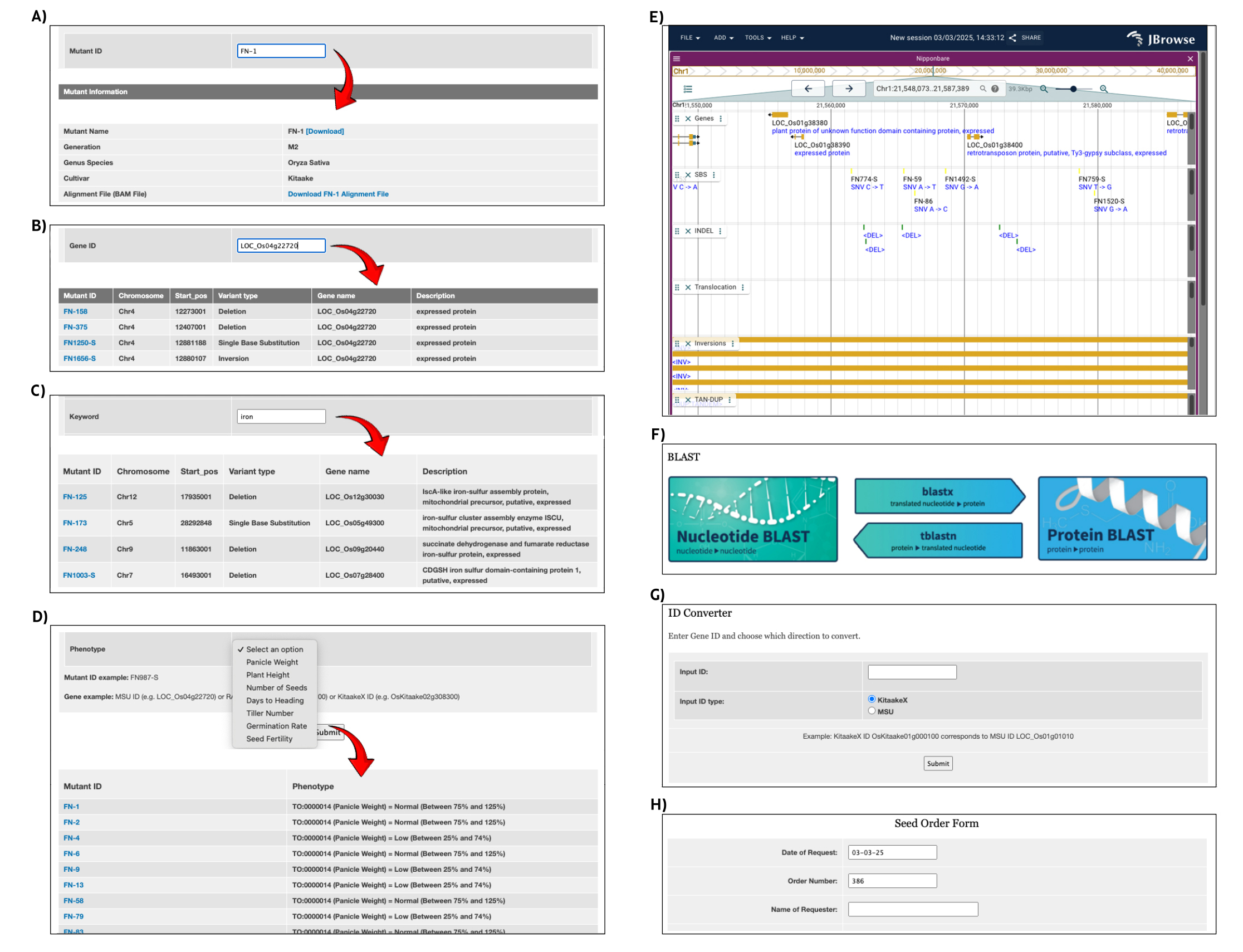

Supplement: baag024_Supplemental_Files [file baag024_supplemental_files.zip › Supplementary_Figure_3.jpg]

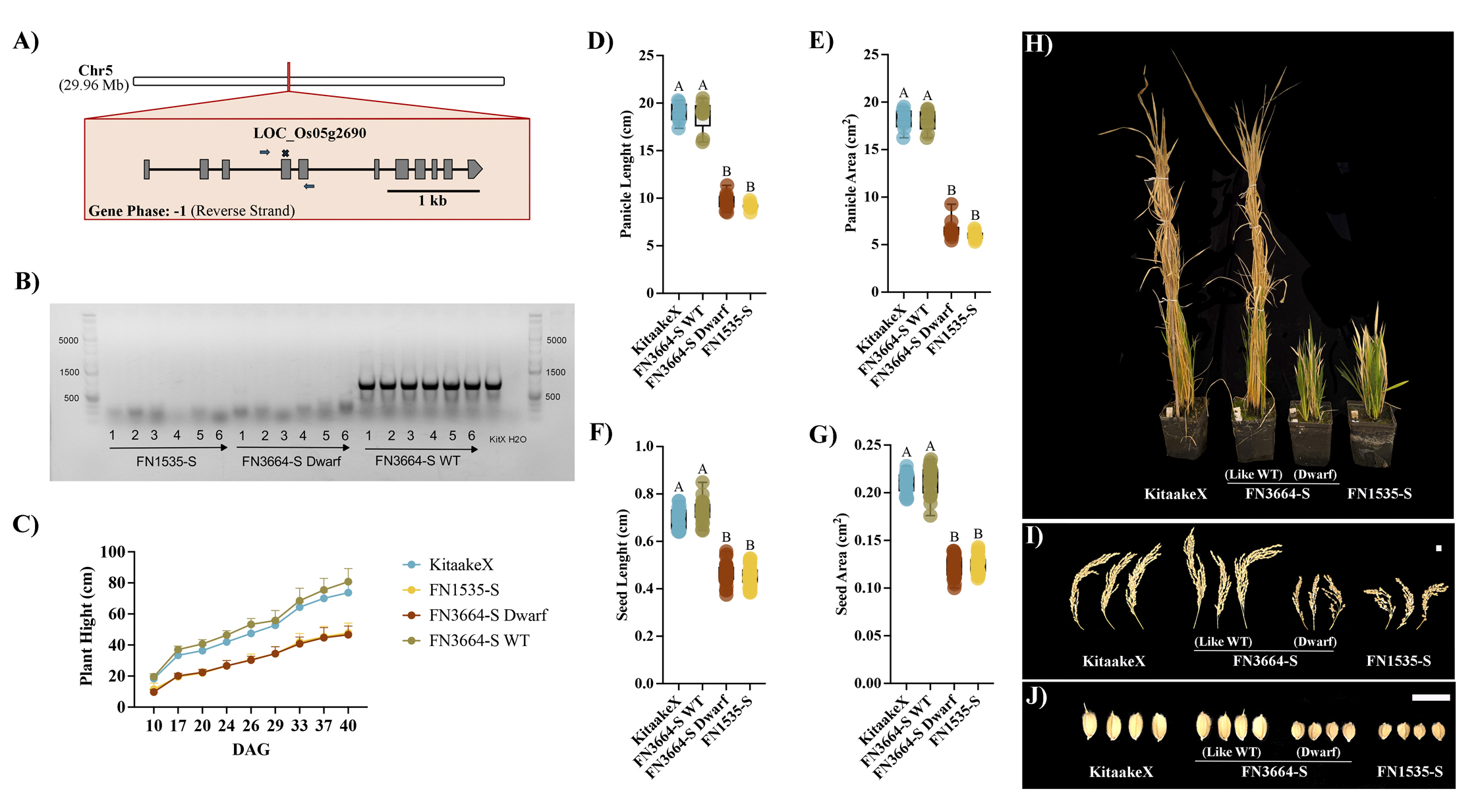

Supplement: baag024_Supplemental_Files [file baag024_supplemental_files.zip › Supplementary_Figure_4_New.jpg]
